# Performance of GPT-4 for automated prostate biopsy decision-making based on mpMRI: a multi-center evidence study

**DOI:** 10.1186/s40779-025-00621-3

**Published:** 2025-07-07

**Authors:** Ming-Jun Shi, Zhi-Xiang Wang, Shuang-Kun Wang, Xuan-Hao Li, Yan-Lin Zhang, Ying Yan, Ran An, Li-Ning Dong, Lei Qiu, Tian Tian, Jia-Xin Liu, Hong-Chen Song, Ya-Fan Wang, Che Deng, Zi-Bing Cao, Hong-Yin Wang, Zheng Wang, Wei Wei, Jian Song, Jian Lu, Xuan Wei, Zhen-Chang Wang

**Affiliations:** 1 Department of Urology, Beijing Friendship Hospital, Capital Medical University, Beijing, 100050 China; 2 Institute of Urology, Beijing Municipal Health Commission, Beijing, 101313 China; 3 Department of Radiology, Beijing Friendship Hospital, Capital Medical University, Beijing, 100050 China; 4 Department of Ultrasound, Beijing Friendship Hospital, Capital Medical University, Beijing, 100050 China; 5 Department of Radiology, Beijing Chaoyang Hospital, Capital Medical University, Beijing, 100020 China; 6 Department of Pathology, Beijing Friendship Hospital, Capital Medical University, Beijing, 100050 China; 7 Department of Urology, Peking University Third Hospital, Beijing, 100083 China; 8 Department of Urology, Beijing Fuxing Hospital, Capital Medical University, Beijing, 100039 China; 9 Department of Urology, Beijing Miyun District Traditional Chinese Medicine Hospital, Beijing, 101500 China; 10 Division of Science and Technology, Beijing Friendship Hospital, Capital Medical University, Beijing, 100050 China

**Keywords:** Prostate biopsy, Generative Pretrained Transformer-4 (GPT-4), Decision-making, Prostate cancer, Multiparametric magnetic resonance imaging (mpMRI)

## Abstract

**Background:**

Multiparametric magnetic resonance imaging (mpMRI) has significantly advanced prostate cancer (PCa) detection, yet decisions on invasive biopsy with moderate prostate imaging reporting and data system (PI-RADS) scores remain ambiguous.

**Methods:**

To explore the decision-making capacity of Generative Pretrained Transformer-4 (GPT-4) for automated prostate biopsy recommendations, we included 2299 individuals who underwent prostate biopsy from 2018 to 2023 in 3 large medical centers, with available mpMRI before biopsy and documented clinical-histopathological records. GPT-4 generated structured reports with given prompts. The performance of GPT-4 was quantified using confusion matrices, and sensitivity, specificity, as well as area under the curve were calculated. Multiple artificial evaluation procedures were conducted. Wilcoxon’s rank sum test, Fisher’s exact test, and Kruskal–Wallis tests were used for comparisons.

**Results:**

Utilizing the largest sample size in the Chinese population, patients with moderate PI-RADS scores (scores 3 and 4) accounted for 39.7% (912/2299), defined as the subset-of-interest (SOI). The detection rates of clinically significant PCa corresponding to PI-RADS scores 2–5 were 9.4, 27.3, 49.2, and 80.1%, respectively. Nearly 47.5% (433/912) of SOI patients were histopathologically proven to have undergone unnecessary prostate biopsies. With the assistance of GPT-4, 20.8% (190/912) of the SOI population could avoid unnecessary biopsies, and it performed even better [28.8% (118/410)] in the most heterogeneous subgroup of PI-RADS score 3. More than 90.0% of GPT-4 -generated reports were comprehensive and easy to understand, but less satisfied with the accuracy (82.8%). GPT-4 also demonstrated cognitive potential for handling complex problems. Additionally, the Chain of Thought method enabled us to better understand the decision-making logic behind GPT-4. Eventually, we developed a ProstAIGuide platform to facilitate accessibility for both doctors and patients.

**Conclusions:**

This multi-center study highlights the clinical utility of GPT-4 for prostate biopsy decision-making and advances our understanding of the latest artificial intelligence implementation in various medical scenarios.

**Supplementary Information:**

The online version contains supplementary material available at 10.1186/s40779-025-00621-3.

## Background

Prostate cancer (PCa) is one of the most common malignancies in men, with an estimated 1.4 million diagnoses and 375,000 deaths worldwide in 2020 [1–3]. Prostate biopsy is the mainstay of PCa diagnosis and key to detecting clinically significant PCa (csPCa, Gleason score 3 + 4 and higher), which requires further active treatment. The need for prostate biopsy is based on elevated prostate-specific antigen (PSA) levels, free-PSA (fPSA)/total-PSA (tPSA) ratio, PSA density (PSAD), suspicious digital rectal exam findings, and/or other biomarkers [4–6]. Additionally, physician–patient shared decision-making is also important [7, 8]. However, unnecessary biopsies are common in clinical management, and over-diagnosis of non-csPCa raises concerns about potential complications, including sepsis [9–11].

With the emergence of multiparametric magnetic resonance imaging (mpMRI), the detection of csPCa before biopsy has shown great promise [12, 13]. The routine use of the evolved Prostate Imaging Reporting and Data System (PI-RADS) has demonstrated its power to support more informed decisions regarding biopsy [14–17]. Particularly, the avoidance of unnecessary prostate biopsy and the detection of csPCa has been largely improved by using MRI-fusion-guided targeted biopsy [18, 19]. This five-score system (on a scale of 1–5) predicts the likelihood of csPCa as low (PI-RADS scores 1 or 2), intermediate (PI-RADS score 3), high (PI-RADS score 4), or very high (PI-RADS score 5) [16], with corresponding probabilities of csPCa at 6, 12, 48, and 72%, respectively [20]. The need for a prostate biopsy lies not only in integrating and evaluating multiple clinical parameters but also in assessing the heterogeneous subset with moderate PI-RADS scores (scores 3 and 4) [21, 22].

Artificial intelligence (AI) has been increasingly applied in various clinical scenarios, particularly in the field of medical imaging [23]. Recently, Generative Pretrained Transformer-4 (GPT-4), a revolutionary AI technology and large language model (LLM), has garnered great interest in medicine. Given its capabilities, GPT-4 may assist in triage and disease diagnosis, mimic physician–patient consultations, analyze and generate medical reports, summarize the key points from extensive literature, and, not least, provide emotional support similar to human partners [24–27]. Considering our situation for proper biopsy recommendation, it is where GPT-4 can play a role—by utilizing a broad knowledge base, analyzing high-dimensional data within seconds, efficiently generating standardized mpMRI reports with consistency, responding in a near-human manner, and ultimately offering sound advice in response to inquiry prompts.

In this multi-center study, we collected a large sample size focused on patients with moderate PI-RADS scores [defined as the subset-of-interest (SOI)] and aimed to test the performance of GPT-4 in providing automated prostate biopsy suggestions.

## Materials and methods

### Study population, clinical variables, and histopathology

This is a multicenter study and data were retrospectively collected from 3 large medical centers: cohort 1, Beijing Friendship Hospital; cohort 2, Beijing Chaoyang Hospital; and cohort 3, Peking University Third Hospital. Overall, 3321 men with suspicious PCa who underwent prostate biopsy between May 2018 and May 2023 were collected in these 3 centers. Considering the mpMRI examination before biopsy, 2299 patients were finally obtained by following exclusions: (1) patients with PCa history; (2) not biopsy-naïve; (3) mpMRI records not within 6 months; (4) no records for PSA level before biopsy or not within 6 months; and (5) no confirmed histopathologic diagnostic records. The flowchart of the patient selection process is presented in Additional file 1: Fig. S1. This study was approved by the Ethics Committee of Beijing Friendship Hospital and shared among multi-centers (2023-P2-240-01).

Standard clinical variables of interest included age, tPSA, fPSA/tPSA ratio, PSAD, and available PCa family history. A 3.0-Tesla MRI (Prisma, Siemens, Erlangen, Germany) were used routinely for every prostate scanning, and our mpMRI included T1-weighted imaging (T1WI), T2-weighted imaging (T2WI), diffusion weighted imaging (DWI) with 6 b values (50, 200, 500, 1000, 1500, and 2000 s/mm^2^, respectively), apparent diffusion coefficient (ADC) mapping and dynamic contrast enhanced (DCE) perfusion for most cases. The PI-RADS v2 was applied for prostate MRI imaging analyses and evaluations. mpMRI variables included PI-RADS scores and descriptive reports of prostate MRI examinations.

A minimum of 12 cores biopsy has been widely accepted as a systematic biopsy and nearly all centers utilized the ultrasound-guided systematic biopsy procedure (ranges from 12 to 24 cores) for most of the included cases. However, our center (cohort 1) nearly systematically adopted transperineal (TP) biopsy while the other two centers conducted a transrectal (TR) approach for the majority of patients. Histopathologic variables included the final pathological diagnosis, categorized as either benign disease or PCa, the Gleason score of malignancy, and the corresponding grade classification (Grade group 1: Gleason score 2–6; Grade group 2: Gleason score 3 + 4 = 7; Grade group 3: Gleason score 4 + 3 = 7; Grade group 4: Gleason score 8; and Grade group 5: Gleason scores 9–10) [28, 29]. Gleason scores and grade groups were reported and csPCa was defined as Gleason score ≥ 3 + 4.

### Risk calculators’ validation within our dataset

The Prospective Loyola University mpMRI (PLUM) [30] and stanford prostate cancer calculator (SPCC) [31] are two newly updated risk calculators (RCs) which considered not only clinical parameters but also PI-RADS scores of mpMRI evaluation. These two RCs are both open access user-friendly websites (https://www.prostatecancer-riskcalculator.com/seven-prostate-cancer-risk-calculators#CalculatorContainer for PLUM and https://med.stanford.edu/ucil/nomogram.html for SPCC). PLUM RC has a few input restrictions: (1) age must be between 50 and 75 years; (2) PSA value must be between 0.4 and 50 ng/ml; and (3) prostate volume must be between 10 and 110 ml.

### GPT-4 generated report

We selected a heterogeneous subset of patients with moderate PI-RADS scores (scores 3 and 4), namely SOI. We input clinical data, descriptive mpMRI reports, and artificial determiners as prompts in the GPT-4 chat session, and then asked GPT-4 to analyze and provide proper biopsy recommendations. The artificial determiners were as follows: (1) advanced age and family history of PCa increase the rate of PCa; (2) tPSA ≤ 4 ng/ml cannot completely exclude PCa; (3) the fPSA/tPSA ratio has less diagnostic value in patients with a tPSA ≥ 10 ng/ml; (4) tPSA value between 4 and 10 ng/ml belongs to the gray zone of PCa diagnosis and needs to further consider the fPSA/tPSA ratio < 0.15 suggests an increased possibility of PCa; (5) PSAD > 0.15 suggests an increased possibility of PCa; (6) 70% of PCa occurs in the peripheral zone, and 20–30% occurs in the migratory zone, the central zone, and other regions; (7) typical MRI features of PCa are low signal nodules in T1WI and T2WI, DWI showing high signal and ADC with low signal and rich blood supply nodules in dynamic enhancement DCE; and (8) PI-RADS evaluation is mainly based on PI-RADS v2.0 or PI-RADS v2.1 version. Brief descriptions regarding the evaluation of GPT-4 performance and GPT-4 decision visualization are shown below, the details are described in Additional file 1: Methods.

### Evaluation of GPT-4 performance

The performance of GPT-4 was analyzed using confusion matrix analyses and by calculating sensitivity, specificity, and area under the curves (AUCs). Benefiting from our large dataset, we also compared this performance with published RCs, such as the PLUM and SPCC. We designed a six-criteria scale for further scoring (accuracy, exhaustivity, intelligibility, practicability, personalization, and compliance), where higher scores represent better performance. Additional relatively challenging questions were designed to explore the “cognitive” limits of GPT-4.

### GPT-4 decision visualization

Chain of Thought (CoT) [32] was applied to trace the decision-making pattern of GPT-4. Two examples of CoT visualization are displayed in Additional file 1: Table S1. To test the reproducibility of GPT-4’s output, we additionally embedded CoT within a prompt, independently generated biopsy recommendation report focusing on the SOI subset, and compared it with that without CoT. A diagnostic platform termed “ProstAIGuide” was developed. It is a user-friendly online diagnostic tool that enables both urologists and patients to obtain preliminary prostate biopsy advice in a fast, reliable, and labor-saving way. Noteworthy, our platform utilized GPT-4 as a priority, other versions such as GPT-3.5 may reduce predictive efficiency.

### Statistical analysis

Statistical analyses were performed using R (version 4.0.0) or GraphPad Prism software (version 9.0.1). Data shown were median (interquartile range, IQR) for descriptive variables. Sensitivity, specificity, and AUCs were calculated. Wilcoxon’s rank sum test, Fisher’s exact test, and Kruskal–Wallis tests were used for the comparisons. A *P-*value < 0.05 in two-tailed tests was considered statistically significant.

## Results

### Baseline characteristics

Their demographic, clinical, PI-RADS groups on mpMRI and histopathologic diagnoses are shown in Table 1. Patients with a PI-RADS score of 1 were pooled together with those with a PI-RADS score of 2. There were 577, 410, 502, and 810 patients corresponding to the 4 groups, with PI-RADS scores of 1–2, 3, 4, and 5, respectively. Among them, SOI patients accounted for 39.7% (912/2299), with proportions of 36.3% (298/820), 49.7% (349/702), and 34.1% (265/777) for cohorts 1, 2, and 3, respectively. The overall number of patients and the proportion of histopathologically proven malignant disorders were comparable among the 3 centers. The overall median tPSA was 11.17 ng/ml and significantly differed among the 4 PI-RADS groups. Approximately 39.9% of individuals received TP biopsy, whereas 58.4% underwent the TR approach.

**Table 1:** Baseline characteristics of the included cohort

Variables/Outcomes	Overall (*n* = 2299)	Subgroups by PI-RADS scores				H-statistic	*P-*value
		PI-RADS score 1–2 (*n* = 577)	PI-RADS score 3 (*n* = 410)	PI-RADS score 4 (*n* = 502)	PI-RADS score 5 (*n* = 810)		
Age [years, median (IQR)]	69 (64–75)	67 (62–72)	68 (63–74)	69 (64–75)	72 (66–78)	23.46	< 0.001
Multi-centers [*n* (%)]							
Cohort 1	820 (35.7)	190 (32.9)	93 (22.7)	205 (40.8)	332 (41.0)		
Cohort 2	702 (30.5)	131 (22.7)	168 (41.0)	181 (36.1)	222 (27.4)		
Cohort 3	777 (33.8)	256 (44.4)	149 (36.3)	116 (23.1)	256 (31.6)		
Clinical [median (IQR)]							
tPSA (ng/ml)	11.17 (6.69–23.06)	9.08 (6.01–13.60)	8.63 (5.89–15.31)	9.13 (5.74–15.92)	22.50 (10.70–71.37)	185.98	< 0.001
fPSA/tPSA ratio	0.16 (0.10–0.57)	0.17 (0.12–0.24)	0.16 (0.11–0.25)	0.15 (0.10–0.20)	0.14 (0.10–0.20)	48.62	< 0.001
PSAD	0.25 (0.13–0.57)	0.17 (0.11–0.26)	0.20 (0.11–0.34)	0.23 (0.12–0.44)	0.56 (0.25–1.36)	454.37	< 0.001
Biopsy path [*n* (%)]							
TP	918 (39.9)	213 (36.9)	121 (29.5)	219 (43.6)	365 (45.1)		
TR	1343 (58.4)	359 (62.2)	280 (68.3)	275 (54.8)	429 (53.0		
Unknown	38 (1.7)	5 (0.9)	9 (2.2)	8 (1.6)	16 (2.0)		
Biopsy protocol [*n* (%)]							
Systematic*	2168 (94.3)	561 (97.2)	391 (95.4)	478 (95.2)	738 (91.1)		
Targeted	128 (5.6)	15 (2.6)	19 (4.6)	22 (4.4)	72 (8.9)		
Pathology [*n* (%)]							
Benign	1011 (44.0)	485 (84.1)	253 (61.7)	180 (35.9)	93 (11.5)		
csPCa	1062 (46.2)	54 (9.4)	112 (27.3)	247 (49.2)	649 (80.1)		

*PSA* prostate-specific antigen, *PSAD* PSA density, *PI-RADS* prostate imaging reporting and data system, *ISUP* International Society of Urological Pathology, *TP* transperineal, *TR* transrectal, *csPCa* clinically significant prostate cancer, represent those of ISUP Gleason scores ≥ 7; Cohort 1 was collected from Beijing Friendship Hospital, Cohort 2 from Beijing Chaoyang Hospital, and Cohort 3 from Peking University Third Hospital; ^*^Systematic biopsy represents biopsy cores ≥ 12, while Targeted biopsy as < 12 cores; H-statistics correspond to Kruskal–Wallis tests for comparisons of continuous variables across 4 PI-RADS subgroups

Of note, nearly all centers utilized ultrasound-guided systematic biopsy procedures (12 to 24 cores) for most included cases (94.3%). As expected, with increasing PI-RADS scores, PCa detection rates increased dramatically. The probabilities of csPCa were 9.4, 27.3, 49.2, and 80.1% for the aforementioned 4 PI-RADS score groups. Notably, 47.5% (433/912) of SOI patients [61.7% (253/410) vs. 35.9% (180/502) for PI-RADS score 3 and score 4, respectively] were histopathologically confirmed to have benign diseases, raising questions about the value of invasive biopsy in these cases.

### GPT-4 generates structured reports

**Fig. 1:**
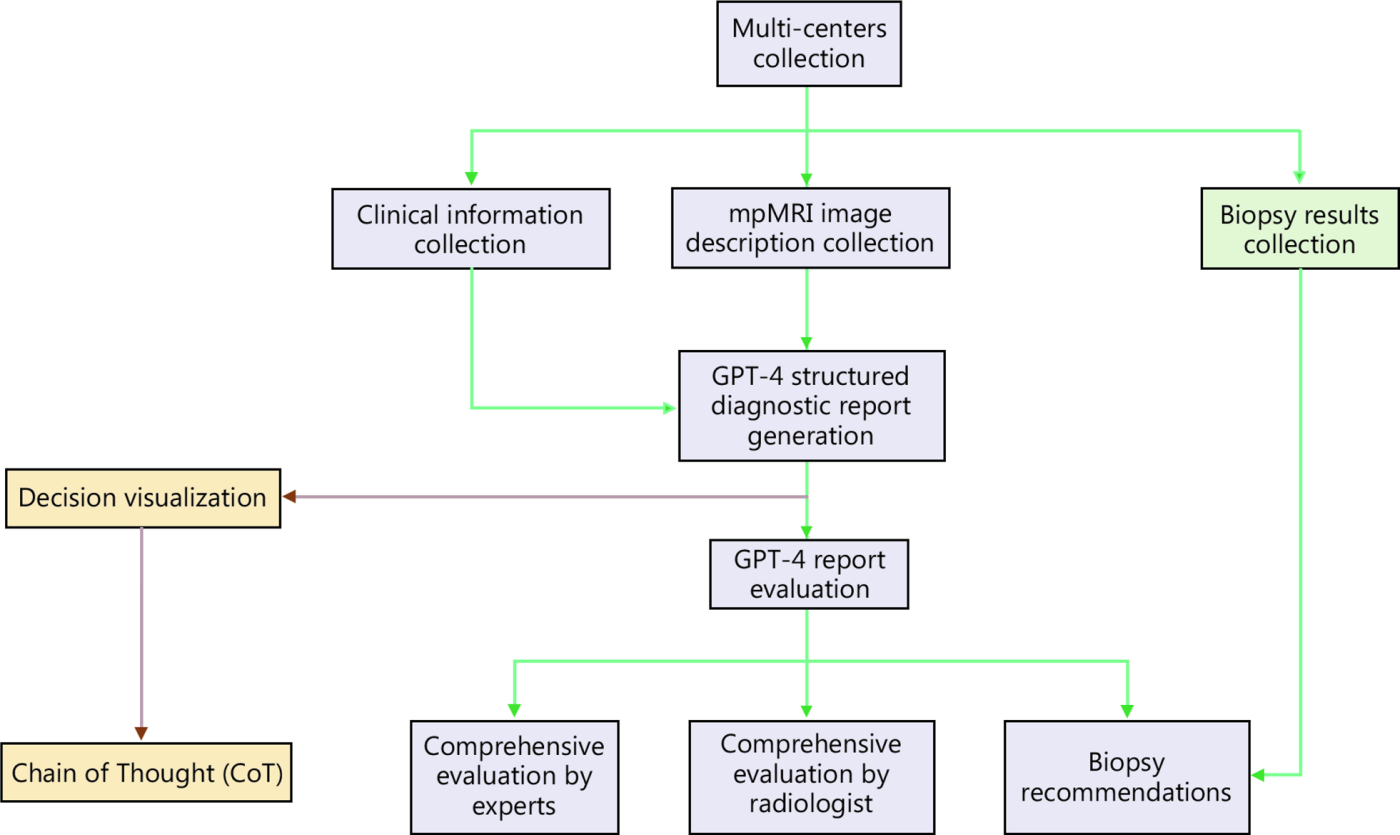
Flowchart indicating Generative Pretrained Transformer (GPT)-4 process track. Biopsy decisions based on the GPT-4-generated report are evaluated in multiple steps and compared with biopsy histopathology

A flowchart illustrating the GPT-4 process is shown in Fig. 1. Two typical examples of our “dialogues” with GPT-4 and the generated reports are displayed in Additional file 1: Table S2. Briefly, for each patient, GPT-4 considered the contributions of age, PSA, PSAD, and lesion characteristics and presentations across different MRI parameters, and then provided a probable PI-RADS score and a likelihood of biopsy necessity.

### GPT-4 shows good performance for biopsy recommendation

**Fig. 2:**
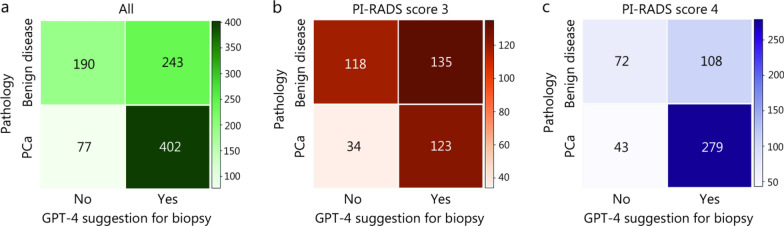
Confusion matrices displaying pathological outcomes vs. Generative Pretrained Transformer (GPT)-4 recommendations for biopsy. **a** Overall population (*n* = 912); **b** Patients with Prostate Imaging Reporting and Data System (PI-RADS) score 3 (*n* = 410); **c** Patients with PI-RADS score 4 (*n* = 502). The pathological outcomes were categorized as either prostate cancers (PCa) or benign diseases

Using pathological diagnoses as the gold standard, we focused on the SOI population and evaluated GPT-4’s performance in providing biopsy recommendations, visualized in confusion matrices (Fig. 2). According to GPT-4’s recommendations, 20.8% (190/912) of the SOI population could avoid unnecessary biopsies (Fig. 2a). Of note, the rates of unnecessary biopsy avoidance were 18.5% (55/298), 23.8% (83/349), and 19.6% (52/265) in cohorts 1, 2, and 3, respectively, suggesting stable performance across centers (Additional file 1: Fig. S2). Remarkably, the probability of biopsy avoidance in SOI patients with PI-RADS score 3 was nearly doubled to that with score 4 [28.8% (118/410) vs. 14.3% (72/502), respectively; Fig. 2b, c].

We further calculated sensitivity, specificity, and AUC to evaluate GPT-4’s diagnostic ability (Additional file 1: Table S3). Overall, GPT-4 achieved a sensitivity of 0.84 but had poor specificity (0.44), and the AUC was 0.65. The false-positive recommendation rates were 32.9% (135/410) and 21.5% (108/502) in the SOI groups with PI-RADS scores 3 and 4, respectively (Fig. 2), indicating that the group with PI-RADS score 3 was the most heterogeneous subgroup.

### Comparison with classical RCs or CoT embedded approach

To evaluate GPT-4’s performance in predicting csPCa, we compared it with the two most popular RCs, PLUM and SPCC, using our large Chinese cohort. Based on PLUM input restrictions, a total of 965 individuals were eligible for comparison. Overall, the two RCs showed similar performance, with the PLUM yielding an AUC of 0.81 (sensitivity = 0.69, specificity = 0.78) and the SPCC achieving an AUC of 0.80 (sensitivity = 0.77, specificity = 0.70) (Table 2). Although our GPT-4 model demonstrated a superior sensitivity of 0.90, it yielded an unsatisfactory AUC of 0.67 and a low specificity of 0.41 (Table 2). Together, compared with real-world practitioners, GPT-4 assistance showed better performance for biopsy recommendations, although there is still room for improvement.

**Table 2:** Performance comparison for csPCa diagnosis among different RCs models using a cohort of 965 patients

Methods/Metrics	AUC	Accuracy	Sensitivity	Specificity
PLUM	0.81	0.73	0.69	0.78
SPCC	0.80	0.74	0.77	0.70
GPT-4	0.67	0.71	0.90	0.41

*csPCa* clinically significant prostate cancer, represent those of International Society of Urological Pathology (ISUP) Gleason scores ≥ 7, *AUC* area under curve, *RC* risk calculator, *PLUM* Prospective Loyola University mpMRI, *SPCC* Stanford Prostate Cancer Calculator

**Fig. 3:**
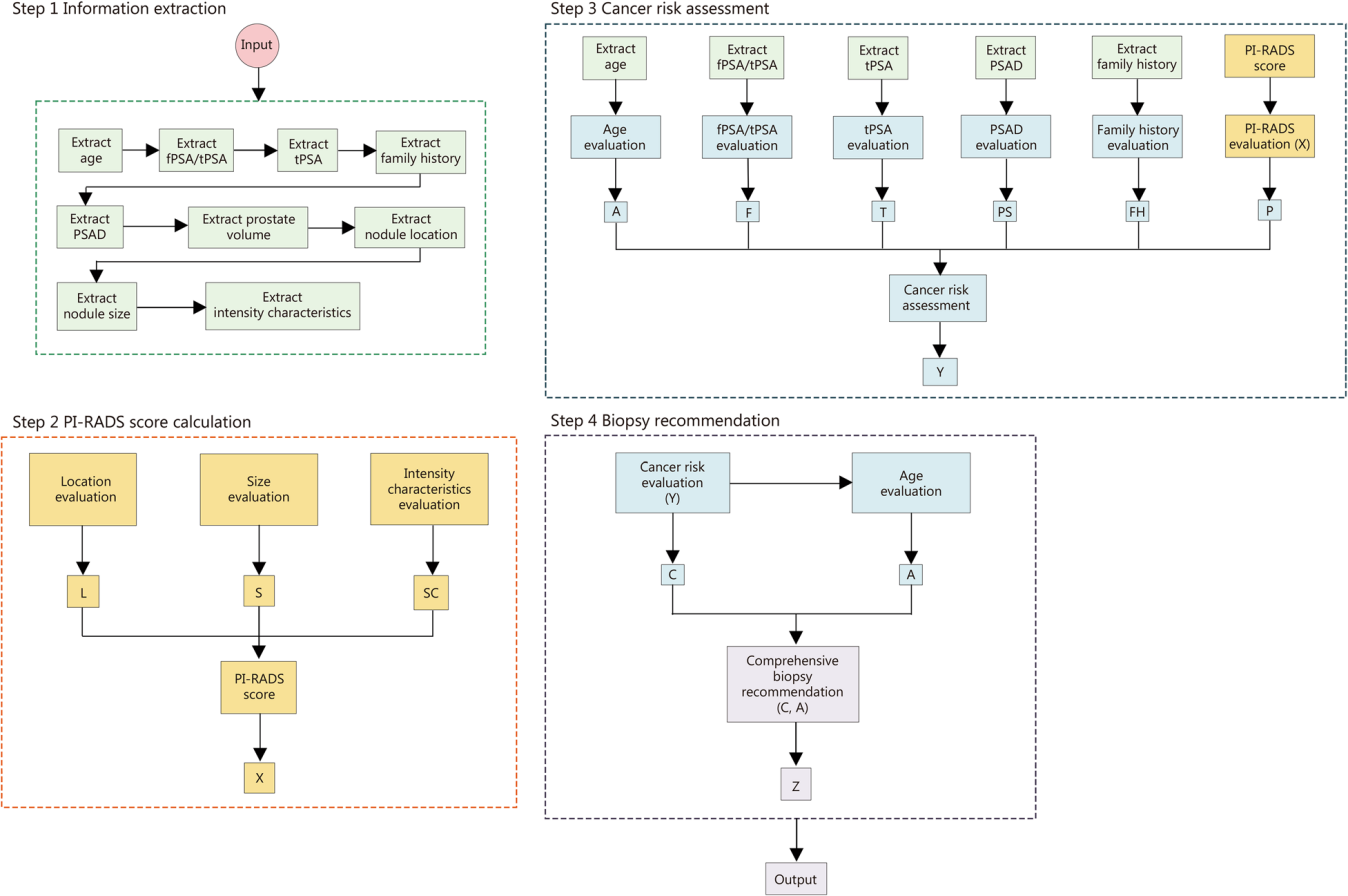
Visualization of the biopsy decision-making process using the Chain of Thought (CoT) approach. The CoT consists of 4 steps: (1) prompts extraction; (2) prostate Imaging Reporting and Data System (PI-RADS) score grouping; (3) likelihood of PCa prediction; and (4) biopsy recommendation. PI-RADS score is represented as X, cancer risk as Y and final biopsy suggestion as Z. These steps form the framework that transparently outlines the logic behind the final recommendation

We also applied the CoT method to visualize GPT-4’s step-by-step decision-making process (Fig. 3). There were 4 key steps in our context: (1) prompt extraction; (2) PI-RADS score grouping; (3) PCa likelihood prediction; and (4) biopsy recommendation. Importantly, multiple factors could be considered either sequentially or concurrently at each step. Some factors, such as age, may influence more than one step. Additionally, we compared GPT-4 generated diagnostic reports with and without the CoT integration and found the proportion of the SOI population that could avoid unnecessary biopsies was similar [22.8% (208/912) vs. 20.8% (190/912), respectively]. However, the embedded CoT method yielded a significantly higher prediction accuracy for biopsy recommendations compared to its counterpart [71.9% (656/912) vs. 64.9% (592/912), *P* < 0.001; Additional file 1: Fig. S3]. Together, our visualization chart clarified the reasoning behind each individual and overall decision step, and a double-check with CoT embedded strategy reflected reproducibility of GPT-4, enabling a better understanding of GPT-4’s decision logic and reinforcing confidence in its use for biopsy recommendations.

### Experts’ inspection for GPT-4 capability

**Fig. 4:**
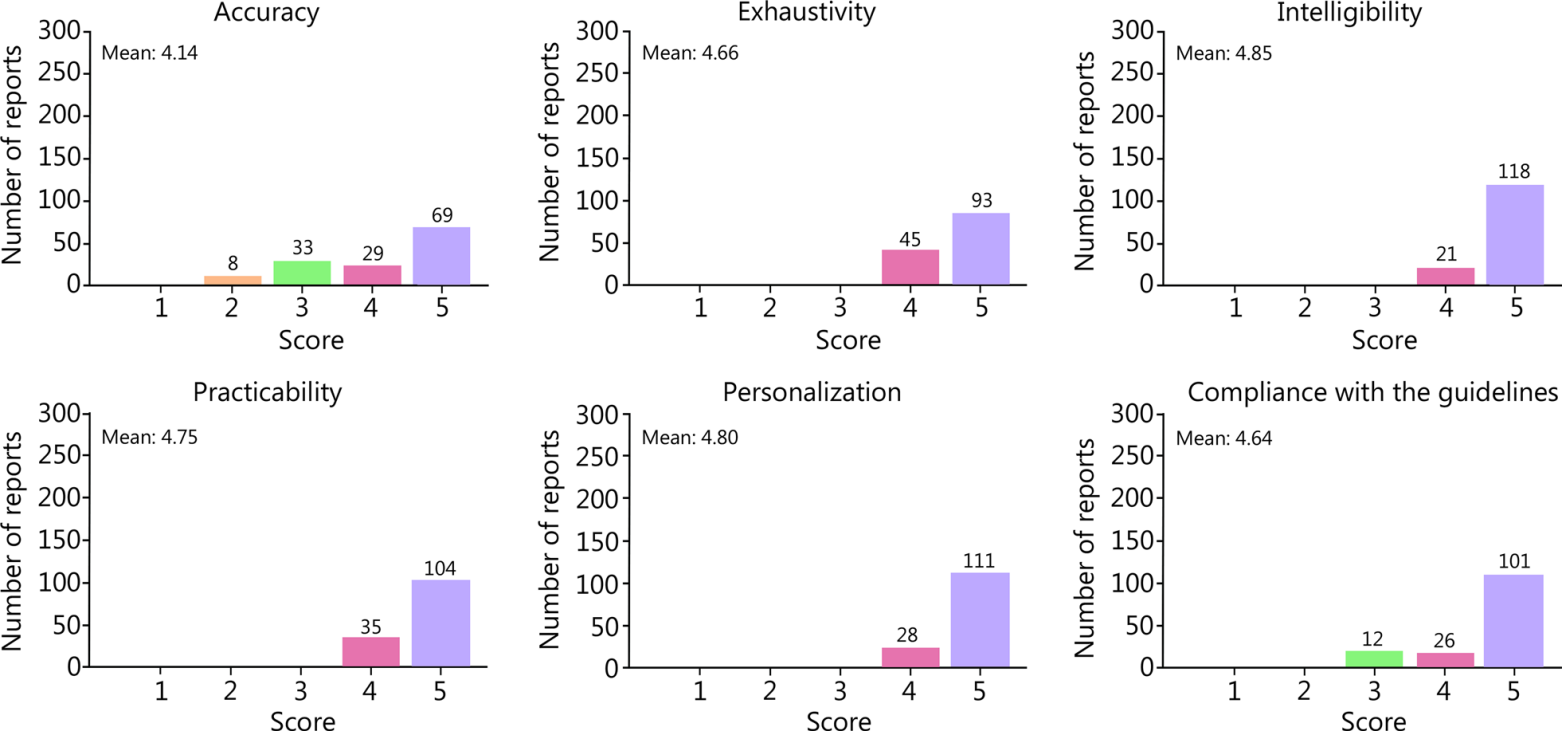
Artificial evaluation of Generative Pretrained Transformer (GPT)-4-generated reports (*n* = 139) using a six-criteria scale. Panels represent accuracy, exhaustivity, intelligibility, practicability, personalization, and guideline compliance. Each criterion was scored from 1 to 5

As reported, GPT-4 can make mistakes and may sometimes provide imaginative answers, which could be dangerous in medical applications [27]. Therefore, a rigorous and important evaluation step is included herein. From the experts’ inspection overview within a sampling subgroup (*n* = 139), more than 90.0% (corresponding to a score of 4.5 out of 5) of GPT-4-generated reports were comprehensive, easy to understand, practical for decision-making, and personalized. However, only 82.8% (4.14/5.0) of the reports were satisfactory regarding accuracy (Fig. 4). This was likely due to inconsistencies between PI-RADS grouping and the predicted diagnosis, implying a fundamental gap between GPT-4 and professional interpretations. To further explore GPT-4’s “cognitive” limits, we selected 3 relatively challenging questions to assess whether they could provide satisfactory responses. The “dialogues” for these questions are shown in Additional file 1: Table S4. Collectively, we found that GPT-4 understood the context well, elaborated arguments in a convincing manner, weighed pros and cons, and even speculated on prospects. In other words, GPT-4 demonstrated promising competence and cognitive potential for handling complex problems.

### ProstAIGuide platform assists in automated biopsy decision-making

We developed an automated diagnostic platform, termed ProstAIGuide, to help urologists alleviate their workload by using this intelligent assistant on a routine basis. Meanwhile, this platform may also assist patients in shared decision-making. Although ProstAIGuide provides preliminary prostate biopsy advice in a fast, reliable, and labor-saving way, additional human evaluation as a double-check is essential in medical settings, as GPT-4 is not perfect, and there is no room for error in medicine. The interface of the ProstAIGuide platform is shown in Additional file 1: Fig. S4. This online tool can be accessed at: http://39.103.60.61:8080/.

## Discussion

To our knowledge, this multi-center study represents the largest sample size for evaluating the probability of PCa predicted using PI-RADS scores. With the upgrading of PI-RADS scores, Barkovich et al. [20] reported that the detection of csPCa dramatically increased, and the probabilities were 6, 12, 48, and 72%, respectively. This was lower than what we found, which were 9.4, 27.3, 49.2, and 80.1% for different PI-RADS score groups. Although patients presenting with a PI-RADS score of 5 are classified as having a very high possibility of csPCa, the true positive rate remains unsatisfactory. There are a few possible reasons accounting for this: (1) some benign diseases resemble PCa on imaging, such as chronic prostatitis, granulomatous prostatitis, or hyperplastic nodules; (2) due to technical factors (MRI artifacts, motion, poor quality) or the radiologist’s subjective interpretation, there might be overestimation on MRI for PI-RADS scoring; and (3) sampling bias or fusion technique in biopsy may have an influence.

Unnecessary prostate biopsy causes both physical and mental harm, sometimes severe, and wastes both medical and economic resources. Despite the successful implementation of mpMRI in prostate disease, it remains difficult to select appropriate patients for biopsy, particularly when dealing with those who have a PI-RADS score of 3. For example, our results showed that approximately 47.5% of SOI patients (61.7% with PI-RADS score 3 and 35.9% with score 4, respectively) underwent unnecessary biopsies.

The MRI-ultrasound software-based fusion method is becoming popular and is believed to significantly improve biopsy precision, but it is still not routinely used in most centers. Instead, targeted biopsy following cognitive fusion with a preoperative mpMRI is recommended due to its slightly higher detection rate for csPCa over systematic biopsy [33]. Interestingly, Pepe et al. [34] recently reported that prior targeted biopsy using ^68^Ga-prostate-specific membrane antigen (PSMA) positron emission tomography/computed tomography further improved the accuracy in the diagnosis of csPCa compared to mpMRI (92.0% vs. 86.2%, respectively). Furthermore, although several high-quality randomized-controlled trials only demonstrated equal or marginally significant advantages for csPCa detection by utilizing TP over TR biopsy in the TRANSLATE, PROBE-PC, PREVENT, and PERFECT trials (60% vs. 54%, 62% vs. 59%, 53% vs. 50%, and 47% vs. 54%, respectively) [35], the findings offer important insights. The TP approach is still recommended because of its proposed advantage of a lower infection risk and improved cancer detection, especially for tumors located at the anterior prostate or apex area [36].

To help eliminate unnecessary biopsies, several RCs have been developed for PCa prediction, such as the early PBCG RC [37], PCPT RC [38], and ERSPC RC [39], which only consider clinical parameters and have overall AUCs below 75%. Orbe Villota et al. [40] recently validated the PBCG RC and ERSPC RC in an Argentinian population (*n* = 250) and found similar performances (0.79 vs. 0.73, respectively). Promisingly, with the evolution of RCs, the PLUM [30] and SPCC [31] are two newly updated models that consider clinical parameters and PI-RADS scores from mpMRI evaluations. These demonstrated superior performances compared to traditional RCs (AUCs ranging from 0.80 to 0.85). Consistently, Massanova et al. [5] also found that the synergistic analysis of clinical and PI-RADS parameters performed well, even in the most heterogeneous group (PI-RADS score of 3). For the first time, we validated these two RCs in a large Chinese population and found similar performance for predicting csPCa (AUC around 0.80).

**Fig. 5:**
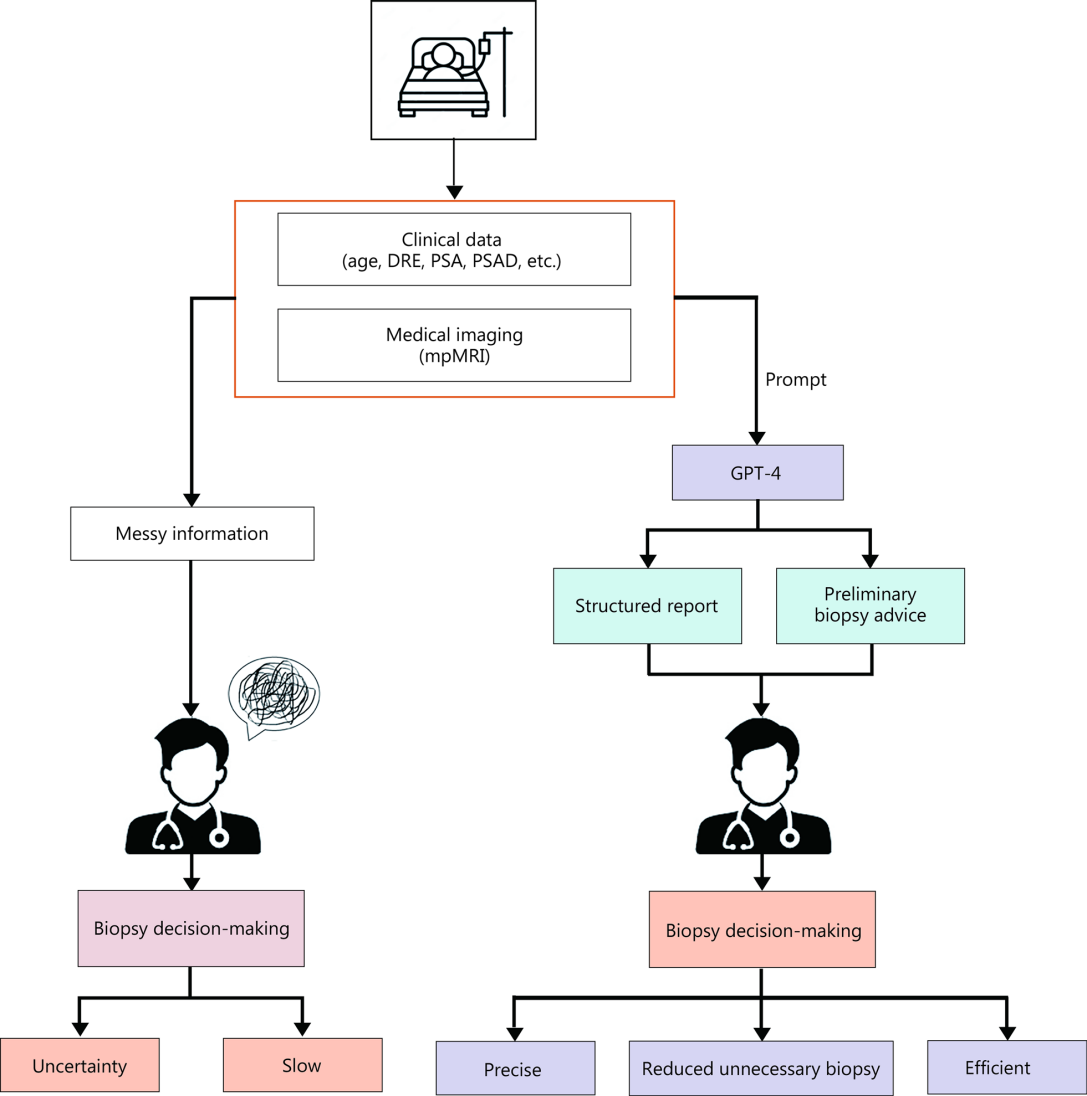
Prostate biopsy decision-making with and without GPT-4. GPT-4 Generative Pretrained Transformer-4, DRE digital rectal exam, PSA prostate-specific antigen, PSAD PSA density, mpMRI multiparametric magnetic resonance imaging

Recently, GPT-4 has gained significant attention for its potential applications in various medical scenarios [24–27]. We tested GPT-4’s performance in automated prostate biopsy decision-making using real-world data and provided preliminary evidence in this field. A comparative workflow is illustrated in Fig. 5. When focusing on SOI patients, we found that 20.8% of the overall group (28.8% with PI-RADS score 3 vs. 14.3% with score 4) could avoid biopsies. This demonstrates the advantage of GPT-4 in our scenario, with even better performance in the most heterogeneous subgroup, PI-RADS score 3. Moreover, considering that the median PSA level in our cohort was higher than in Western datasets used for the PLUM or SPCC models (11.17 ng/ml vs*.* 6.3 ng/ml or 7.6 ng/ml, respectively), GPT-4 may have overestimated cancer risk within our cohort and might perform better in more appropriately matched populations. This may also explain the relatively low specificity (0.41) of our GPT-4 predictive model. Regrettably, we observed inferior performance from GPT-4 compared to PLUM or SPCC, implying GPT-4’s current limitations and the need for improvement in LLMs for such medical scenarios. Notably, the evaluation process used by GPT-4 was less affected by individual subjectivity, provided advantages in accuracy and other criteria, and was transparent and rational, as illustrated through CoT visualization. Importantly, the ProstAIGuide platform we developed was easy to use for both doctors and patients, implying significant potential for clinical value. Altogether, we demonstrated the success, strengths, and reliability of GPT-4 for prostate biopsy triage and offered an example of how its potential could be extended to broader medical applications.

To minimize any potential medical harm, we should be mindful of GPT-4—recommended decisions and avoid unusual errors like “hallucination” [27, 41]. Currently, there are few approaches to assess GPT-4—generated output. Instead, we designed a stepwise process with relatively strict procedures to evaluate the performance of GPT-4. Experts’ inspection based on a six-criteria scale and selected advanced questions revealed that GPT-4 performed excellently in extracting massive evidence from the literature, interpreting and integrating extensive medical data, handling problems with great complexity, and providing practical and personalized options. Furthermore, one remarkable highlight of our study is the implementation of the recently developed CoT method to partially unveil the “black box” of AI-relevant decision-making processes [42]. This significantly facilitated transparency and clarity, enhanced interpretability, and rationalized the decision logic.

With the development of AI, the discovery of task-specific radiomics features related to cancer diagnosis has shown great promise. Several studies have investigated deep learning and radiomics-based features to predict PCa, achieving AUCs ranging from 0.70 to 0.90 [43–45]. Additionally, genetic testing is becoming increasingly popular for early PCa detection due to its strong hereditary component, estimated to contribute 5–15% of cases [46]. For example, the mutations in homologous recombination repair genes (*BRCA1/2*, *ATM*, and *CHEK2*) and mismatch repair genes (*MLH1*, *MSH2*, *PMS2*, and *MSH6*) are associated with varying degrees of increased predisposition to PCa, leading to recommended genetic testing in different clinical guidelines [47, 48]. Meanwhile, liquid biopsy based on circulating tumor DNA [49] and genome-wide polygenic risk scores [50] have also rapidly developed to personalize PCa screening. Consequently, making appropriate decisions based on all these high-dimensional data is more than a brainstorming exercise, it is nearly impossible to achieve with a simple calculator or single-task model. Hopefully, future LLMs capable of analyzing complex multi-modality images and integrating clinical data, and PI-RADS scores, and genetic testing outputs will better support clinical decision-making.

The present study has several limitations that should be acknowledged. First, the predictive accuracy of GPT-4 needs to be improved, especially regarding the PI-RADS score 3 group, where both professionals and GPT-4 face challenges. One plausible solution depends on the competence of next generation GPT, which could analyze medical imaging directly and integrate high-dimensional data from various modalities. Second, GPT-4 is principally pretrained with English text and is less relevant to Chinese prompts. Consequently, our results may have a potential bias since we used Chinese prompts throughout the analysis. Given the ongoing development of GPT, this concern may be alleviated soon. Lastly, there are hidden issues regarding medical safety, individual privacy, and ethnicity, which prevent us from conducting a prospective study design. Despite these limitations, the study introduced GPT-4 as a solution for a critical clinical question and provided compelling evidence for its utility.

## Conclusions

Avoiding unnecessary prostate biopsies is a critical issue in routine medical management. We focused on a specific and heterogeneous population subset, took advantage of GPT-4, and validated its utility in aiding prostate biopsy decision-making, especially its apparent advantage in the most heterogeneous subgroup, PI-RADS score 3. We also developed a user-friendly platform–ProstAIGuide–facilitating automated prostate biopsy decision-making. Overall, incorporating urgent clinical needs and the latest AI innovations, our results demonstrated good performance by GPT-4 for prostate biopsy triage and paved a new path for its potential implementation in other medical scenarios.

## Supplementary Information

**Additional file 1. Methods. Fig. S1** Flowchart of patient screening, exclusions, and eligibility. **Fig. S2** Subgroup analysis of confusion matrices showing pathological outcomes vs. Generative Pretrained Transformer (GPT)-4 biopsy recommendations across 3 cohorts. **Fig. S3** Confusion matrices displaying pathological outcomes vs. Generative Pretrained Transformer (GPT)-4 recommendations for biopsy (*n* = 912). **Fig. S4** Interface of the PostAIGuide platform. **Table S1** Two examples of Chain of Thought (CoT) responses. **Table S2** Two typical examples of “dialogues” with Generative Pretrained Transformer (GPT)-4 and the generated reports. **Table S3** Quantitative results from the confusion matrix. **Table S4** Three relatively challenging questions and GPT-4’s responses.

## Data Availability

All mpMRI imaging descriptive reports and GPT-4 generated reports in Chinese were available on requirement.

## Abbreviations

ADC: Apparent diffusion coefficient
AI: Artificial intelligence
AUCs: Area under the curves
COT: Chain of thought
csPCa: Clinically significant prostate cancer
DCE: Dynamic contrast enhanced
DWI: Diffusion weighted imaging
fPSA: Free-PSA
GPT-4: Generative Pretrained Transformer-4
LLM: Large language model
mpMRI: Multiparametric magnetic resonance imaging
PCa: Prostate cancer
PI-RADS: Prostate imaging reporting and data system
PLUM: Prospective Loyola University mpMRI
PSA: Prostate-specific antigen
PSAD: PSA density
PSMA: Prostate-specific membrane antigen
RC: Risk calculator
SOI: Subset-of-interest
SPCC: Stanford prostate cancer calculator
tPSA: Total-PSA
TP: Transperineal
TR: Transrectal
T1WI: T1-weighted imaging
T2WI: T2-weighted imaging
