# Supplementary material for: Performance of GPT-4 for automated prostate biopsy decision-making based on mpMRI: a multi-center evidence study
Source: Mil Med Res. 2025 Jul 7;12:33. doi: 10.1186/s40779-025-00621-3 (PMC12232764; doi:10.1186/s40779-025-00621-3)
Supplement: Supplementary file 1 — Additional file 1. Methods. Fig. S1 Flowchart of patient screening, exclusions, and eligibility. Fig. S2 Subgroup analysis of confusion matrices showing pathological outcomes vs. Generative Pretrained Transformer (GPT)-4 biopsy recommendations across 3 cohorts. Fig. S3 Confusion matrices displaying pathological outcomes vs. Generative Pretrained Transformer (GPT)-4 recommendations for biopsy (n = 912). Fig. S4 Interface of the PostAIGuide platform. Table S1 Two examples of Chain of Thought (CoT) responses. Table S2 Two typical examples of “dialogues” with Generative Pretrained Transformer (GPT)-4 and the generated reports. Table S3 Quantitative results from the confusion matrix. Table S4 Three relatively challenging questions and GPT-4’s responses. [file 40779_2025_621_MOESM1_ESM.pdf]

Methods

Assessment of reliance on Generative Pretrained Transformer-4 (GPT-4)

Taking pathologic diagnosis as standard, we evaluated the performance of GPT-4, represented by biopsy recommendation outcome, by conducting confusion matrix analyses and calculating sensitivity, specificity, and area under the curves (AUCs). Consequent subgroup analyses were stratified for 4 groups of Prostate Imaging Reporting and Data System (PI-RADS) score 1 – 2, PI-RADS score 3, PI-RADS score 4, and PI-RADS score 5, respectively.

To give an overall evaluation for GPT-4 generated reports, we designed a six-criteria-based scale and invited 6 experienced specialists (3 radiologists and 3 urologists, all with over 10 years of working experience) for further scoring (accuracy, exhaustivity, intelligibility, practicability, personalization, compliance). Each of these criteria is on a 5-point scale, with higher scores representing better performance of GPT-4. The 6 criteria and definitions are shown below: 1) accuracy: to what extent GPT-4 correctly report the relevance of clinical features, the lesions properties, and PI-RADS groups, and the accordance with histopathology; 2) exhaustivity: to what extent GPT-4 provide a comprehensive and detailed report; 3) intelligibility: to what extent GPT-4 provides an easy-to-understand report; 4) practicability: to what extent GPT-4 generated report is helpful for biopsy decision-making; 5) personalization: to what extent GPT-4 provides personalized or humanized advice; and 6) compliance with the guidelines: to what extent GPT-4 generated report is approximate to clinical guidelines or best practices.

Lastly, to further evaluate the depth of GPT-4 for mastering knowledge related to prostate cancer (PCa), we invited urology experts to propose 3 relatively challenging and multi-faceted questions to explore the "cognitive" limit of GPT-4. The evaluation system is in line with the aforementioned six-criteria-based scales. The 3 questions were as follows: 1) What's the standard for advanced age in the context relevant to PCa diagnosis? 2) Since the PSA level and PI-RADS scores were two main factors contributing to the final biopsy decision, how to weigh when a patient presents a low PSA but a high PI-RADS score and vice versa? 3) Both MRI reports indicate patients bearing obvious metastatic PCa, why was one recommended for biopsy while the other was not?

Chain of Thought (CoT) and GPT-4 decision visualization

The CoT is a newly developed method that facilitates reasoning and transparent explainability in large

language models (LLMs). To better understand the GPT-4 decision process, we applied the CoT method to decompose step by step, visualized in a flowchart, and could trace the decision pattern of GPT-4. It improves the interpretability and increases the convincing force for suggestions provided by GPT-4. A detailed explanation of the CoT method and its efficacy in promoting GPT-4 transparency is provided below.

The dissection of the CoT for GPT-4 output analysis necessitates several steps, including the formulation of a symbolic language, the definition of operators, and the design of reasoning steps.

Symbolic language

In this task, it is imperative to define certain symbols that represent key information in the report and the operations that we need to perform. The symbols we have defined are as follows. 1) Age: represents the age of the patient. 2) free-prostate-specific antigen (fPSA)/total-prostate-specific antigen (tPSA) ratio: represents the ratio of free to total PSA. 3) tPSA: represents the total PSA value. 4) Prostate volume: represents the volume of the prostate. 5) PSA density (PSAD): PSA density, represents the ratio of tPSA to the volume of the prostate. 6) Nodule location: represents the location of the nodule. 7) Nodule size: represents the size of the nodule. 8) Intensity characteristics: represents the intensity characteristics of the nodule.

Operators

We need to define certain operators that represent the operations we need to perform. The operators we have defined are as follows. 1) Extract (X): represents the operation of extracting the value of X. For example, “Extract (Age)” represents the extraction of age, “Extract (fPSA/tPSA ratio)” represents the extraction of the fPSA/tPSA ratio.

Reasoning steps

We need to define some complex symbols to represent our reasoning steps. The reasoning steps we have defined are as follows. 1) PI-RADS score (nodule location, nodule size, intensity characteristics): represents the operation of assigning a PI-RADS score based on the location, size, and intensity characteristics of the nodule. 2) Cancer risk assessment (age, fPSA/tPSA ratio, tPSA, PSAD, PI-RADS score): represents the operation of assessing the risk of cancer based on age, fPSA/tPSA ratio, tPSA value, PSAD, and PI-RADS score. 3) Biopsy recommendation (cancer risk assessment): represents the operation of providing a recommendation for biopsy based on the result of the cancer risk assessment.

This document clearly defines our symbolic language, operators, and reasoning steps, enabling GPT-4 to better understand our task. Two input and output examples for the CoT are shown in the **Additional file 1: Table S1**.

To validate the reproducibility of GPT-4's output, we applied the CoT method as an independent control experiment. We focused on subset-of-interest (SOI) patients (*n* = 912) and developed two independent prompting strategies: 1) A direct instruction prompt that asks GPT-4 to provide structured diagnostic results; 2) An additional CoT-embedded prompt that asks GPT-4 to provide the same format diagnostic report. We then compared these two methods in terms of model reproducibility and diagnostic accuracy.

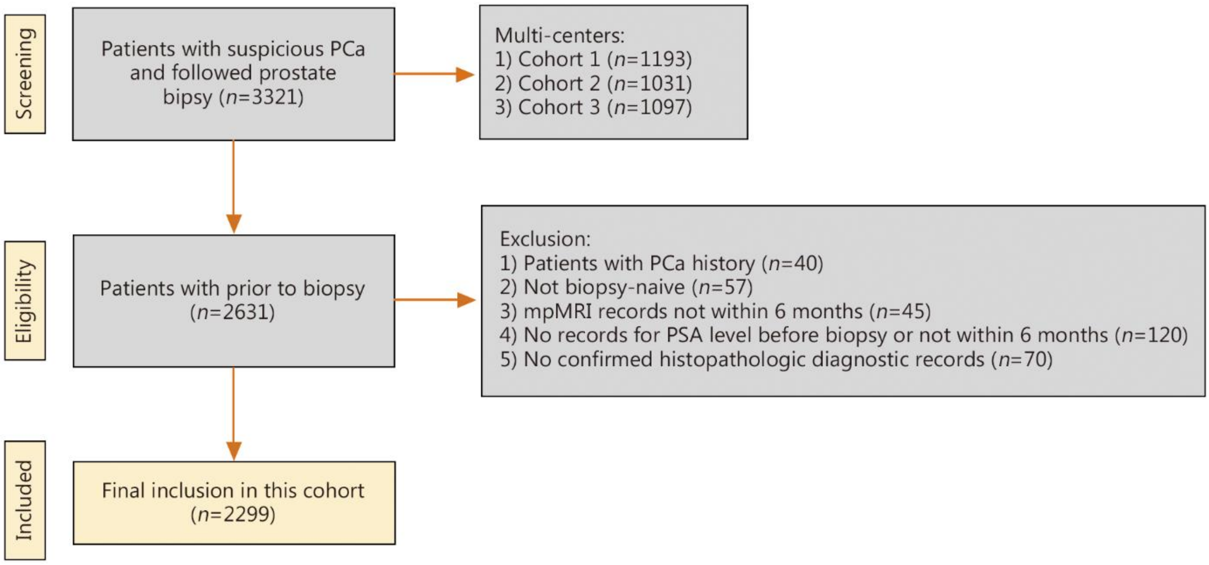

Fig. S1 Flowchart of patient screening, exclusions, and eligibility. PCa prostate cancer, mpMRI multiparametric magnetic resonance imaging, PSA prostate-specific antigen

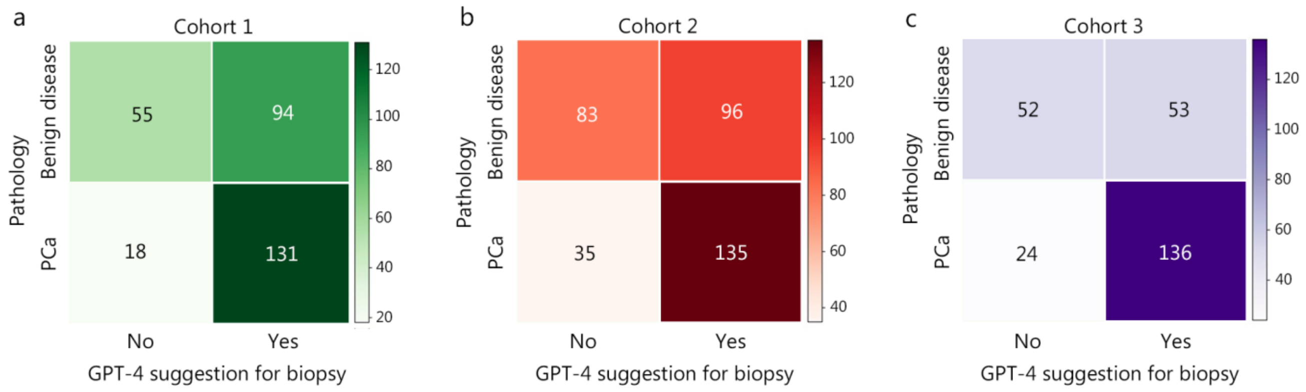

Fig. S2 Subgroup analysis of confusion matrices showing pathological outcomes vs. Generative Pretrained Transformer (GPT)-4 biopsy recommendations across 3 cohorts. **a** Cohort 1 – Beijing Friendship Hospital (*n* = 298); **b** Cohort 2 – Beijing Chaoyang Hospital (*n* = 349); **c** Cohort 3 – Peking University Third Hospital (*n* = 265). PCa prostate cancer

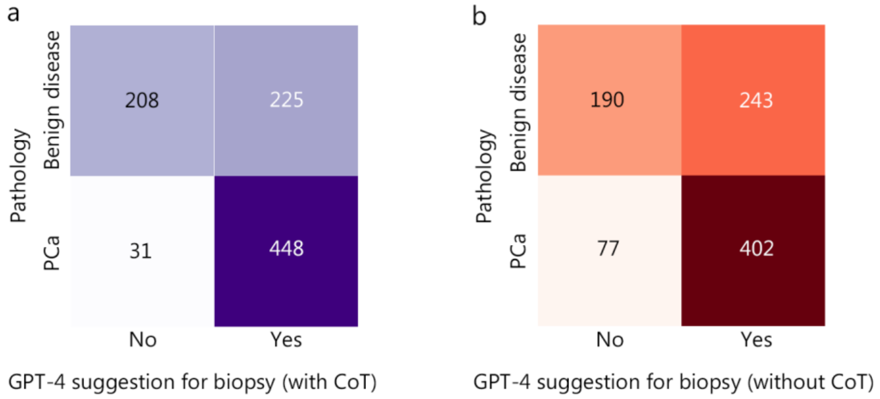

Fig. S3 Confusion matrices displaying pathological outcomes vs. Generative Pretrained Transformer (GPT)-4 recommendations for biopsy (*n* = 912). **a** Prompt without Chain of Thought (CoT); **b** Prompt with CoT integration. The pathological outcomes were categorized as either prostate cancers (PCa) or benign diseases

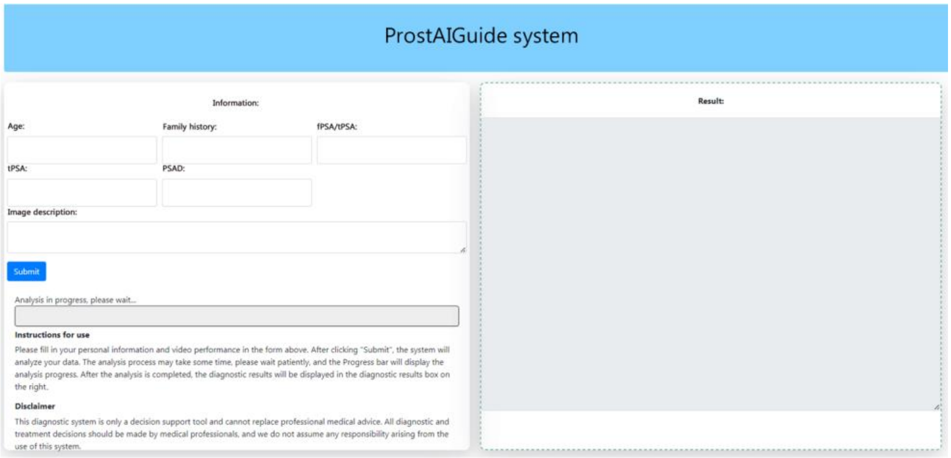

Fig. S4 Interface of the PostAIGuide platform. Generative Pretrained Transformer (GPT)-4 is the preferred model; using earlier versions like GPT-3.5 may reduce predictive performance

Table S1 Two examples of Chain of Thought (CoT) responses

Cases	Age (years)	Family history	fPSA/tPSA ratio	tPSA (ng/ml)	PSAD	mpMRI description	CoT decomposition	Histopathology results
Case1	73	No	0.45	0.87	0.045	The volume of the prostate is slightly enlarged, and the size is about 2.4 cm × 4.2 cm × 3.7 cm (anterior-posterior diameter × left-right diameter × upper-bottom diameter). The transition zone is enlarged, the local shape is plump, and multiple nodules can be seen inside, showing high and low mixed intensities on T2WI, surrounded by low intensities around, and enhanced scanning shows uneven enhancement. Slightly to the right of the transitional zone at the bottom of the prostate, abnormal intensity nodules were seen, with a long diameter of 1.0 cm. T2WI showed relatively uniform low intensity with clear borders, DWI showed high intensity, ADC intensity decreased, and enhanced scan arterial phase was significantly enhanced. In the peripheral	Here is thought chain for the diagnosis task:<br>1) Information extraction. ① Extract age () → 73. In this step, the patient's age information is extracted from the medical record. ② Extract fPSA/tPSA ratio () → 0.45. The fPSA/tPSA ratio is extracted from the medical record, which is an indicator helpful for distinguishing between PCa and benign prostatic hyperplasia. ③ Extract tPSA () → 0.87. The tPSA value is extracted, which is an important indicator of PCa. ④Extract prostate volume () → 2.4 cm × 4.2 cm × 3.7 cm. Information about the prostate volume is extracted. ⑤ Extract family history () → No. ⑥ Extract PSAD () → 0.045. ⑦ Extract nodule location () → "At the base of the prostate transition zone slightly to the right", "in the central part of the posterior prostate". The location of the nodules is extracted. ⑧ Extract nodule size () → 1.0 cm, 0.5 cm. The size of the nodules is extracted. ⑨ Extract intensity characteristics () → "Uniformly low intensity on T2WI", "high intensity on DWI", "reduced ADC intensity". MRI intensity characteristics information is extracted.<br>2) PI-RADS score calculation. ① Location evaluation ("at the base of the prostate transition zone slightly to the right") → L1. The location of the	Benign hyperplasia

Cases	Age (years)	Family history	fPSA/tPSA ratio	tPSA (ng/ml)	PSAD	mpMRI description	CoT decomposition	Histopathology results
						zone of the prostate, a patchy area of decreased intensity can be seen on T2WI, and the enhanced scan shows delayed enhancement. A water-like intensity shadow with a diameter of about 0.5 cm can be seen in the center of the posterior part of the prostate, and the capsule can be seen at the edge, and the enhanced scan shows delayed enhancement of the capsule. There is no abnormal high intensity in the prostate on DWI. The prostate capsule is intact. The shape and size of the bilateral seminal vesicles were normal, and the intensity was normal. The bladder is well filled, the wall is not thick, and there is no abnormality in the cavity. There were no enlarged lymph nodes in the bilateral pelvic wall and groin area. There was no clear intensity abnormality in the pelvic components	nodule is initially evaluated. ② Size evaluation (1.0 cm) → S1. The size of the nodule is evaluated. ③ Intensity characteristics evaluation ("uniformly low intensity on T2WI", "high intensity on DWI", "reduced ADC intensity") → SC1. The MRI intensity characteristics of the nodule are evaluated. ④ PI-RADS score (L1, S1, SC1) → X. The PI-RADS score is calculated based on the above information.<br>3) Cancer risk assessment. ① Age evaluation (73) → A1. The patient's age is evaluated. ② fPSA/tPSA evaluation (0.45) → F1. The fPSA/tPSA ratio is evaluated. ③ tPSA evaluation (0.87) → T1. The tPSA value is evaluated. ④ Family history evaluation (0) → FH. The family history is evaluated. ⑤ PSAD evaluation (0.045) → PS. The PSAD is evaluated. ⑥ PI-RADS evaluation (X) → P1. The PI-RADS score is evaluated. ⑦ Cancer risk assessment (A1, F1, T1, FH, PS, P1) → Y. The cancer risk is assessed by integrating the above information.<br>4) Biopsy recommendation. ① Cancer risk evaluation (Y) → C1. The cancer risk is evaluated. ② Age evaluation (73) → A1. The patient's age is re-evaluated. ③ Comprehensive biopsy recommendation (C1, A1) → Z. Based on the above information, a recommendation for a biopsy is given.<br>This detailed thought chain covers the entire process from information extraction to the final diagnostic	

Cases	Age (years)	Family history	fPSA/tPSA ratio	tPSA (ng/ml)	PSAD	mpMRI description	CoT decomposition	Histopathology results
							recommendation. Each step has a corresponding output that will be used for the next evaluation. However, it should be noted that this thought chain is a basic framework, the actual diagnostic and treatment process may need to consider more factors and need to be executed by an experienced doctor	
Case 2	69	No	0.046	44.83	1.531	The prostate volume is enlarged, measuring approximately 4.2 cm × 3.1 cm × 4.3 cm (superoinferior diameter × anteroposterior diameter × transverse diameter). Multiple mixed high and low intensities shadows are seen in the transitional zone of the prostate on T2WI, showing heterogeneous enhancement changes. The low intensity ring around the transitional zone is incomplete. Abnormal patchy intensities are seen on both sides of the peripheral zone of the prostate (especially on the right side) and the right side of the transitional zone. The lesion presents low intensity on T2WI, high intensity on DWI, and low intensity changes on ADC. The	Based on the information you provided, I will analyze this case step by step. Below is the thought chain I constructed based on your question and the specific information of the patient: 1) Information extraction. ① Extract age () → 69. Extract the patient's age from the medical records. ② Extract fPSA/tPSA () → 0.046. Extract the fPSA/tPSA ratio, which is a helpful indicator to differentiate between PCa and benign prostatic hyperplasia. ③ Extract tPSA () → 44.83. Extract the tPSA value, a crucial indicator of PCa. ④ Extract family history () → No. ⑤ Extract PSAD () → 1.531. ⑥ Extract prostate volume () → 4.2 cm × 3.1 cm × 4.3 cm. Extract the information on prostate volume. ⑦ Extract nodule location () → "Primarily located in the transitional zone of the prostate", "both sides of the peripheral zone of the prostate (right side is notable), right side of the transitional zone". Extract the location information of the nodules. ⑧ Extract nodule size () → 3.5 cm. Extract the size of the nodule. ⑨ Extract intensity characteristics () → "Mixed high and low intensity shadow on T2WI",	PCa

Cases	Age (years)	Family history	fPSA/tPSA ratio	tPSA (ng/ml)	PSAD	mpMRI description	CoT decomposition	Histopathology results
						lesion shows significant enhancement changes during the arterial phase of the enhanced scan, with a long diameter of about 3.5 cm. The prostate capsule is incomplete, and the lesion invades the bladder wall anteriorly and superiorly, the seminal vesicle gland posteriorly, and the adjacent neurovascular bundles on both sides are thickened and enhanced. The bladder is inadequately filled, with a thickened wall. No enlarged lymph nodes are seen on both sides of the pelvic wall and the groin area. No clear intensity abnormalities are seen in the bones that make up the pelvis	"showed low intensity on T2WI, high intensity on DWI, and low intensity change on ADC". Extract the information on MRI intensity characteristics.<br>2) Calculating PI-RADS score. ① Location evaluation ("primarily located in the transitional zone of the prostate", "both sides of the peripheral zone of the prostate (right side is notable), right side of the transitional zone") → L1, L2. Make a preliminary evaluation of the location of the nodules. ② Size evaluation (3.5 cm) → S1. Evaluate the size of the nodules. ③ Intensity characteristics evaluation ("mixed high and low intensity shadow on T2WI", "showed low intensity on T2WI, high intensity on DWI, and low intensity change on ADC") → SC1, SC2. Evaluate the MRI intensity characteristics of the nodules. ④ PI-RADS score (L1, S1, SC1) → X1. Calculate the PI-RADS score for the first nodule based on the above information. ⑤ PI-RADS Score (L2, S1, SC2) → X2. Calculate the PI-RADS score for the second nodule based on the above information.<br>3) Assessing cancer risk. ① Age evaluation (69) → A1. Evaluate the patient's age. ② fPSA/tPSA ratio evaluation (0.046) → F1. Evaluate the fPSA/tPSA ratio. ③ tPSA evaluation (44.83) → T1. Evaluate the tPSA value. ④ Family history evaluation (0) → FH. The family history is evaluated. ⑤ PSAD evaluation (1.531) → PS. The PSAD is evaluated. ⑥ PI-RADS evaluation (X1) → P1. Evaluate the PI-	

Cases	Age (years)	Family history	fPSA/tPSA ratio	tPSA (ng/ml)	PSAD	mpMRI description	CoT decomposition	Histopathology results
							RADS score for the first nodule. ⑦ PI-RADS evaluation (X2) → P2. Evaluate the PI-RADS score for the second nodule. ⑧ Cancer risk assessment (A1, F1, T1, FH, PS, P1, P2) → Y. Combine the above information to assess the risk of cancer.<br>4) Biopsy Recommendation. ① Cancer risk evaluation (Y) → C1. Evaluate the risk of cancer. ② Age evaluation (69) → A1. Re-evaluate the patient's age. ③ Comprehensive biopsy recommendation (C1, A1) → Z.<br>Based on the above information, provide a recommendation for biopsy.<br>This detailed thought chain covers the entire process from information extraction to the final diagnostic recommendation. Each step has a corresponding output, which will be used for the next evaluation. However, it should be noted that this thought chain is just a basic framework, and the actual diagnosis and treatment process may need to consider more factors, and need to be performed by experienced doctors	

PCa prostate cancer, *T2WI* T2-weighted imaging, *DWI* diffusion weighted imaging, *ADC* apparent diffusion coefficient, *fPSA* free prostate-specific antigen, *tPSA* total prostate-specific antigen, *PSAD* prostate-specific antigen density, *mpMRI* multiparametric magnetic resonance imaging, *PI-RADS* Prostate Imaging-Reporting and Data System

Table S2 Two typical examples of "dialogues" with Generative Pretrained Transformer (GPT)-4 and the generated reports

Cases	Age (years)	Family history	tPSA (ng/ml)	fPSA/ tPSA	PSAD	mpMRI descriptive reports	GPT-4 generated reports	Histopathology results
Case 1	69	No	10.47	0.12	0.289	The prostate is enlarged, measuring approximately 35 mm × 44 mm × 45 mm (superior-inferior diameter × anterior-posterior diameter × left-right diameter). Abnormal intensity is seen in the posterior region of the bilateral peripheral zone of the prostate, with slightly low intensity on T2WI, isointense on T1WI, no obvious intensity increases on DWI, mixed isointense and slightly low intensity on ADC, and delayed enhancement on enhanced scanning. A nodule with abnormal intensity, measuring about 0.5 cm × 0.5 cm, is seen at the 4 o'clock position of the left peripheral zone of the prostate, showing isointense on T1WI, isointense to slightly low intensity on T2WI, isointense to slightly high intensity on DWI, no clear low intensity on ADC map, and obvious enhancement in the early phase of enhanced scanning. The transition zone is slightly enlarged, with multiple nodules seen inside, showing mixed high and low intensity on T2WI, surrounded by low intensity, and uneven enhancement on enhanced scanning. A patchy abnormal intensity is seen in the	Based on the patient's clinical information, laboratory test results, and MRI imaging description, we can make the following analysis:<br>1) The patient is 69 years old, which is considered elderly and a risk factor for PCa. However, the patient has no family history of PCa, which is a factor that reduces risk.<br>2) The patient's tPSA value is 10.47 ng/ml, exceeding the normal upper limit of 4 ng/ml, indicating a potential for PCa. Additionally, the patient's fPSA/tPSA ratio is 0.12, which is less than 0.15, further suggesting the possibility of PCa. The patient's PSAD is 0.289, greater than 0.15, also indicating a possible PCa risk.<br>3) According to the patient's MRI imaging description, there are abnormal signals in the posterior peripheral zone of both sides of the prostate and the anterior transition zone on the right side, which are typical locations for PCa. An abnormal signal nodule at the 4 o'clock position of the left peripheral zone of the prostate shows an iso-signal on T1WI, an iso-to-slightly low signal on T2WI, an iso-to-slightly high signal on DWI, no clear low signal on the ADC map, and significant enhancement in the early phase of contrast-enhanced scanning. These are	PCa

Cases	Age (years)	Family history	tPSA (ng/ml)	fPSA/ tPSA	PSAD	mpMRI descriptive reports	GPT-4 generated reports	Histopathology results
						anterior region of the right transition zone of the prostate, showing relatively low intensity on T2WI, isointense to low intensity on T1WI, increased intensity on DWI, relatively low intensity on ADC, and uneven early enhancement on enhanced scanning. The boundary of the lesion is unclear, measuring 1.9 cm × 1.2 cm. The bilateral seminal vesicles are normal in shape and size, and no abnormal intensity is seen. The bladder is well filled, the wall is not thick, and no abnormalities are seen in the cavity. No enlarged lymph nodes are seen on both sides of the pelvic wall and in the groin area. No obvious intensity abnormality is seen in the pelvic bones	typical MRI features of PCa. Therefore, this nodule's PI-RADS score should be 4, indicating a high probability of PCa<br>Considering all the above information, the patient's risk of PCa should be assessed as high. Therefore, I recommend a prostate biopsy to obtain pathological evidence and further confirm the diagnosis	
Case 2	63	No	8.95	0.18	0.105	The prostate is enlarged, measuring approximately 53 mm × 56 mm × 55 mm (superior-inferior diameter × anterior-posterior diameter × left-right diameter). The transition zone is significantly enlarged, with multiple nodules seen inside, showing mixed high and low intensities on T2WI, surrounded by low intensities, and uneven enhancement on enhanced scanning. The lesion compresses the bladder anteriorly. A slightly low-density spot is seen at the apex of the prostate, with a diameter of about 0.7	Based on the patient's clinical information, the patient is 63 years old with no family history of PCa. The advanced age is a risk factor for PCa, but the absence of a family history mitigates this risk, making these two factors effectively cancel each other out.<br>From the laboratory test results, the patient's tPSA value is 8.95 ng/ml, which falls within the 4 – 10 ng/ml gray zone for PCa diagnosis. However, the fPSA/tPSA ratio is 0.18, greater than 0.15, which somewhat reduces the likelihood of PCa. The PSAD value is 0.105, less	Bengin disease

Cases	Age (years)	Family history	tPSA (ng/ml)	fPSA/ tPSA	PSAD	mpMRI descriptive reports	GPT-4 generated reports	Histopathology results
						cm, blurred edges, slightly high intensity on DWI, and slightly low intensity on ADC.<br>No obvious abnormal intensity is seen in the peripheral zone of the prostate. The prostate capsule is intact.<br>The bilateral seminal vesicles are normal in shape and size, and no abnormal intensity is seen. The bladder is partially filled, the wall is not thick, and no abnormalities are seen in the cavity. No enlarged lymph nodes are seen on both sides of the pelvic wall and in the groin area. No obvious intensity abnormality is seen in the pelvic bones	than 0.15, also decreasing the probability of PCa.<br>From the MRI imaging, there is an enlargement of the prostate gland with a significant increase in the transition zone and multiple nodules visible, all of which could potentially indicate PCa. However, the patchy slightly low-density shadow in the apex of the prostate, slightly high signal on DWI, and slightly low signal on ADC are not typical signs and cannot confirm PCa. No significant abnormal signals are observed in the peripheral zone of the prostate, further reducing the likelihood of PCa. According to the PI-RADS v2.1, these imaging features may correspond to a PI-RADS score of 3, indicating a medium probability of PCa.<br>Taking all the information into account, the likelihood of the patient having PCa is moderate. Although the patient's tPSA value is in the diagnostic gray zone for PCa, both the fPSA/tPSA ratio and PSAD value decrease the probability of PCa. The MRI imaging is also not definitive for PCa. Therefore, I recommend a prostate biopsy for the patient to further confirm the diagnosis	

PCa prostate cancer, *T1WI* T1-weighted imaging, *T2WI* T2-weighted imaging, *DWI* diffusion weighted imaging, *ADC* apparent diffusion coefficient, *fPSA* free prostate-specific antigen, *tPSA* total prostate-specific antigen, *PSAD* prostate-specific antigen density, *mpMRI* multiparametric magnetic resonance imaging, *PI-RADS* Prostate Imaging-Reporting and Data System

Table S3 Quantitative results from the confusion matrix

Metrics	Overall	Subgroups by PI-RADS scores		Subgroups by multi-centers		
		Score 3	Score 4	Cohort 1	Cohort 2	Cohort 3
**Sensitivity**	0.84	0.78	0.87	0.88	0.79	0.85
**Specificity**	0.44	0.47	0.40	0.37	0.46	0.50
**AUC**	0.65	0.59	0.70	0.62	0.62	0.71
**Accuracy**	0.64	0.62	0.63	0.62	0.63	0.67

PI-RADS Prostate Imaging Reporting and Data System, *AUC* area under curve, Cohort 1 was collected from Beijing Friendship Hospital, Cohort 2 from Beijing Chaoyang Hospital, and Cohort 3 from Peking University Third Hospital

Table S4 Three relatively challenging questions and GPT-4's responses

**Questions**	**Responses of GPT-4**
**Question 1:** What's the standard for advanced age in the context relevant to PCa diagnosis?	PCa is a common malignant tumor in men, and its incidence is highly related to age. Generally, the incidence of PCa is very low in men under 40, and it significantly increases in men over 50.<br>There is no universal standard for the definition of "elderly", but in medical literature, people aged 65 or 70 and above are often considered as "elderly" or "older" population. According to data from the National Cancer Institute (NCI) in the United States, the median age at diagnosis for PCa is 66, implying that most PCa patients are above this age.<br>In the context of PCa, "elderly" might refer to an even older age, such as over 75 or 80, mainly because the incidence of PCa is especially high in these age brackets. Moreover, age significantly influences the choice of treatment and prognosis for PCa. For instance, for some older people, particularly those with severe comorbidities, monitoring rather than active treatment might be a better option, as PCa usually progresses slowly, and treatments can carry side effects.<br>In conclusion, although the definition of "elderly" is not fixed in medicine, in the context of PCa, patients aged 70 or 75 and above are usually considered "elderly". However, this primarily depends on the specific clinical situation and research context. If there are any doubts, consulting a medical professional is recommended
**Question 2:** Since the PSA level and PI-RADS scores were two main factors contributing to the final biopsy decision, how to weigh when a patient presents a low PSA but high PI-RADS score and vice versa?	PSA is a protein found in the blood, and its levels may rise in the presence of PCa. However, PSA may also increase due to other reasons such as prostatitis or benign prostatic hyperplasia. Therefore, PSA testing has high sensitivity but lower specificity, meaning it may produce false-positive results. A study on PSA sensitivity found that the sensitivity of PSA testing ranges from approximately 21% to 91%, depending on the "cut-off" or diagnostic threshold for PSA levels.<br>PI-RADS, on the other hand, is a system to evaluate prostate MRI images and predict the risk of PCa. PI-RADS is graded from 1 to 5, with higher grades indicating a greater likelihood of PCa. It is reported that the PI-RADS scoring system has high sensitivity and specificity for detecting clinically significant PCa. Some studies have shown that the sensitivity and specificity of a PI-RADS score of 4 or 5 are between 88% – 97% and 39% – 94%, respectively. This high sensitivity and specificity are because PI-RADS not only considers a single biomarker, but also a combination of various radiological features.<br>In summary, both PSA and PI-RADS are important tools in the diagnosis of PCa, but their comparison in sensitivity varies with different clinical situations and patient characteristics. In practice, these two tools are often used in conjunction, improving diagnostic accuracy. In cases of high PSA values but lower PI-RADS scores, further diagnostic steps may be needed to confirm the presence of cancer. Conversely, if PSA values are normal but PI-RADS scores are high, further evaluation may be necessary.<br>This information is based on knowledge up to 2021, and new research or clinical guidelines may change this situation. It is recommended that medical professionals be consulted before making any diagnostic or treatment decisions

Questions	Responses of GPT-4
**Question 3:** Both of the two MRI reports indicate patients bearing obvious metastatic PCa, why was one recommended for biopsy while the other was not?	From the MRI reports and related information for these two patients, we can observe several key differences that might explain why one patient was recommended for a biopsy while the other was not:<br>1) Patient 1 (age: 75 years old)<br>Prostate size and lesions: a smaller prostate with multiple nodular abnormal signals, including tumor invasion into surrounding structures and distant metastases.<br>Other imaging findings: included bilateral seminal vesicles with low intensity marked thickening of the bladder's posterior wall, multiple enlarged lymph nodes in the pelvic and groin area, etc.<br>Biopsy recommendation: biopsy is not recommended. Due to the patient's high risk and possible invasion of surrounding structures and distant metastases, a biopsy may not change the treatment plan. Additionally, recovery might be slower for a 75-year-old patient, and a biopsy could present complications.<br>2) Patient 2 (age: 83 years old)<br>Prostate size and lesions: increased volume of the prostate, involving the entire left peripheral zone, right peripheral zone, bottom of the prostate, left transition zone at the tip of the prostate, left transition zone in the middle of the prostate, and bilateral seminal vesicles. The prostate capsule was incomplete.<br>Other imaging findings: many small lymph nodes in the bilateral pelvic wall, with the largest short diameter of 0.6 cm. The left ischium, right pubic bone, and left sacrum were partially seen with patchy DWI high signal areas.<br>Biopsy recommendation: the report does not specifically mention whether a biopsy is recommended, but a conjecture can be made based on the information provided.<br>Overall comments:<br>Patient 1's condition seems more complex, involving more extensive metastases and invasion. Given his high-risk features and potential complications, he might be directly moved to systemic treatments such as hormonal therapy and chemotherapy, bypassing a biopsy.<br>Patient 2's condition, although also presenting as metastatic PCa, appears to be more localized and lacks some of the severe features mentioned in Patient 1. Therefore, for Patient 2, a biopsy might still be a viable option to more accurately determine the tumor's type and grading, thereby selecting the best targeted treatment plan.<br>It's worth noting that these explanations are primarily based on the information provided in the reports, and clinical decisions usually involve more complex factors including the patient's overall health, life expectancy, existing comorbidities, etc. The final decision should be made by the treatment team after a comprehensive assessment of the patient's condition

GPT Generative Pretrained Transformer, *PCa* Prostate cancer, *PSA* prostate-specific antigen, *MRI* magnetic resonance imaging, *PI-RADS* Prostate Imaging-Reporting and Data System, *DWI* diffusion weighted imaging
